# Directional, Low‐Energy Driven Thermal Actuating Bilayer Enabled by Coordinated Submolecular Switching

**DOI:** 10.1002/advs.202102077

**Published:** 2021-10-23

**Authors:** Michael Leveille, Xinyuan Shen, Wenxin Fu, Ke Jin, Muharrem Acerce, Changchun Wang, Jacqueline Bustamante, Anneka Miller Casas, Yuan Feng, Nien‐Hui Ge, Linda S. Hirst, Sayantani Ghosh, Jennifer Qing Lu

**Affiliations:** ^1^ Physics University of California, Merced Merced 95343 USA; ^2^ Materials Science and Engineering University of California, Merced Merced 95343 USA; ^3^ Macromolecular Science Fudan University Shanghai 200433 P. R. China; ^4^ Chemistry University of California, Irvine Irvine 92697 USA

**Keywords:** conformational change, dibenzocycloocta‐1,5‐diene, negative thermal expansion, thermal actuator, thermal energy harvesting

## Abstract

The authors reveal a thermal actuating bilayer that undergoes reversible deformation in response to low‐energy thermal stimuli, for example, a few degrees of temperature increase. It is made of an aligned carbon nanotube (CNT) sheet covalently connected to a polymer layer in which dibenzocycloocta‐1,5‐diene (DBCOD) actuating units are oriented parallel to CNTs. Upon exposure to low‐energy thermal stimulation, coordinated submolecular‐level conformational changes of DBCODs result in macroscopic thermal contraction. This unique thermal contraction offers distinct advantages. It's inherently fast, repeatable, low‐energy driven, and medium independent. The covalent interface and reversible nature of the conformational change bestow this bilayer with excellent repeatability, up to at least 70 000 cycles. Unlike conventional CNT bilayer systems, this system can achieve high precision actuation readily and can be scaled down to nanoscale. A new platform made of poly(vinylidene fluoride) (PVDF) in tandem with the bilayer can harvest low‐grade thermal energy and convert it into electricity. The platform produces 86 times greater energy than PVDF alone upon exposure to 6 °C thermal fluctuations above room temperature. This platform provides a pathway to low‐grade thermal energy harvesting. It also enables low‐energy driven thermal artificial robotics, ultrasensitive thermal sensors, and remote controlled near infrared (NIR) driven actuators.

## Graphical abstract

A bilayer in which actuating submolecular units are aligned undergoes reversible and controlled shape changes in response to low‐energy stimuli. It is inherently reversible, operating on polymer thermal contraction originating from a submolecular conformational change. Combined with a piezoelectric film, the bilayer's ultra‐sensitivity to temperature variations is exploited for low‐grade thermal energy harvesting.

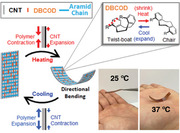

## 1 Introduction

Stimuli responsive polymeric materials that can change shape in response to external stimuli such as humidity,^[^ ^1^, ^2^, ^3^, ^4^, ^5^, ^6^ ^]^ pH,^[^ ^7^ ^]^ solvents,^[^ ^8^, ^9^ ^]^ electric current,^[^ ^3^, ^10^, ^11^, ^12^ ^]^ light,^[^ ^13^, ^14^, ^15^, ^16^ ^]^ or temperature^[^ ^11^, ^15^ ^]^ have been exploited for a large variety of applications including actuators,^[^ ^17^, ^18^, ^19^, ^20^, ^21^ ^]^ microfluidic valves,^[^ ^22^ ^]^ electronic muscles,^[^ ^23^ ^]^ power generators,^[^ ^5^, ^24^ ^]^ biomedical devices,^[^ ^25^, ^26^, ^27^ ^]^ drug delivery,^[^ ^26^, ^27^ ^]^ memory and logic devices,^[^ ^28^, ^29^ ^]^ and more.^[^ ^2^, ^16^, ^30^, ^31^, ^32^ ^]^ The performance of conventional solid‐state systems, independent of solvent and moisture, typically relies on thermodynamic transitions (glass transition, melting, or liquid crystal order‐to‐disorder transitions) and suffer from limited cycle stability due to large‐scale molecular motions that inevitably lead to fatigue.

Emerging molecular and submolecular‐level shape changes which can be used for actuation depend on either a chemical or electrochemical reaction^[^ ^33^ ^]^ or photoinduced *trans‐cis* isomerization or cyclization enabled by bond breaking and reforming.^[^ ^34^, ^35^ ^]^ The former only operates in a specific solution whereas the latter requires a high‐energy stimulus (e.g., ultraviolet light) which is harmful to the surrounding environments (e.g., biological species) and the material itself. A significant number of technologies require low‐energy driven actuating components that operate under an ambient, solid‐state environment, thus highlighting the need for a new system.

We have published a series of papers about a cross‐linked polyarylamide (PAAM) polymer containing dibenzocycloocta‐1,5‐diene (DBCOD) units, PAAM‐DBCOD.^[^ ^36^, ^37^, ^38^ ^]^ DBCOD can be viewed as an eight‐membered, flexible ring fused to two rigid phenyl rings on opposite sides of PAAM chains. The DBCOD unit acts as a molecular switch undergoing a thermally induced conformational change from twist‐boat to chair.^[^ ^39^, ^40^ ^]^ In our previous publications, we have shown that submolecular shape changes of randomly oriented DBCODs in the polymer led to observed giant polymer isotropic thermal contraction.^[^ ^36^, ^37^, ^38^, ^40^, ^41^, ^42^ ^]^ The macroscopic shape change resulting from submolecular conformational changes does not require a solution‐based chemical reaction, high‐energy input to break a covalent bond, nor does it involve large‐scale molecular motion. This enables the creation of low‐energy driven and highly reversible solid‐state actuators.

A bilayer design, consisting of a stimuli responsive layer paired with a passive layer that is insensitive to an external stimulus is a popular approach to construct an actuator that does not require a complex control system.^[^ ^6^, ^43^, ^44^, ^45^, ^46^, ^47^, ^48^, ^49^, ^50^ ^]^ For instance, carbon allotropes such as graphene or carbon nanotubes (CNTs), which have coefficients of thermal expansion (CTEs) of 0–10 ppm K^−1^,^[^ ^51^, ^52^ ^]^ have been combined with a layer of polydimethylsiloxane (PDMS)^[^ ^6^, ^11^, ^20^, ^53^, ^54^ ^]^ or wax,^[^ ^55^ ^]^ which offer large CTE values, to generate mechanical bending due to thermal induced built‐in stress. A carbon layer also offers the ability to produce heat from infrared light absorption, and thus, near infrared (NIR) light can be used as a stimulus for remote actuation.^[^ ^18^ ^]^

In this paper, we describe a new type of bilayer made of ≈14 µm thick PAAM‐DBCOD that is covalently connected to a thin sheet of aligned multiwalled CNTs, with less than 0.1 µm in thickness. The DBCOD conformational change in response to a low‐energy thermal stimulus, such as heating a few degrees above room temperature, can produce an appreciable shape change. Under the same temperature range, the CNTs do not change in shape, thus generating substantial stress. Without any process optimization, ≈4.7 mm per centimeter deflection was observed by heating the film from 25 to 55 °C. A flat bilayer at room temperature (25 °C) placed onto the palm of a hand (37 °C) curled up substantially, further exemplifying the ultrasensitive nature of this new system. Actuator performance has been quantified by bending angle, curvature, and cycle stability. However, since bending performance depends on key factors such as thickness, aspect ratio, modulus, energy input, and energy conversion efficiency,^[^ ^56^, ^57^ ^]^ it is difficult to draw fair comparisons to existing actuators in the literature that often rely on a thermodynamic transition involving large scale molecular motion.

Moreover, moisture available in ambient conditions offers the polymer a greater response compared to dry conditions. Absorbing a small amount of moisture can conceivably plasticize polymer crystalline domains and thus facilitate the DBCOD conformational change. This may have contributed to the significant bending observed when placing a bilayer onto one's palm. Analyses, thermomechanical and open circuit voltage using poly(vinylidene fluoride) (PVDF) as a stress sensor, indicate that actuation can be produced by the DBCOD conformational change only. The presence of moisture allows the occurrence of additional conformational changes.

*π*–*π* stacking between PAAM and CNTs directs assembly of DBCOD actuating units along the longitudinal direction of CNTs, therefore enabling controlled anisotropic shape changes by the design of a CNT pattern. Further, because of covalent linkages between CNTs and polymer chains as well as the intrinsic reversibility of the DBCOD conformational change, this bilayer acts as one, offering excellent cycle performance without any observed changes after 70 000 cycles.

This type of actuator differs from liquid crystal based thermal actuators, moisture‐driven thermal actuators, and other existing thermodynamic‐based actuators in a number of ways. First, the shape change is due to a low‐energy driven conformational change. Second, shape change produced by a submolecular conformational change does not involve large and unrestrained molecular motions such as those at glass transition temperatures and melting temperatures. It therefore offers intrinsically high reversibility and excellent cycle stability. Third, all the submolecular shape changing units are well aligned with respect to each other and to the underlying CNTs. In a synchronized manner, each submolecular shape‐changing event occurs simultaneously, potentiating a macroscopic event. Fourth, the bilayer bending direction is opposite to conventional polymer‐carbon allotrope bilayers which rely on the large thermal expansion of a polymer.

Furthermore, compared with other bilayers, CNTs have an additional two functionalities in the PAAM‐CNT bilayer. Covalent linkages between the CNTs and responsive polymer layer contribute to the bilayer's cycle stability, whereas most published bilayer systems do not offer a covalently reinforced interface. The aligned CNT sheet also serves as a structural guiding layer to direct molecular self‐assembly. As a result, the concerted submolecular conformational changes can be manifested into a substantial macroscopic change. Compared to published thermal actuating systems driven by heat, humidity, NIR, or electrical stimuli, thermal contraction due to collective submolecular events offers distinct advantages and has never been reported before.

Indeed, thermal contraction can be generated by moisture evaporation with increased temperature. However, this moisture‐driven actuation requires precise control of moisture content and thus prohibits its use in many applications. Moreover, a large shape change requires moisture to diffuse into and out of the solid bulk and thus limits the response rate. In comparison, conformational change‐based contraction does not require the presence of moisture and is inherently fast and reversible at the molecular level.^[^ ^39^ ^]^

To demonstrate the efficacy of this unique system, PAAM‐CNT bilayer was coupled with PVDF to serve as an AC generator which has the potential to harness low‐grade waste heat or for ultra‐sensitive thermal sensors. Our environment is replete with waste heat. It can be converted to useful electricity using either thermoelectric or pyroelectric effects.^[^ ^58^, ^59^, ^60^ ^]^ The former requires a large temperature gradient and a complex material system that offers low thermal conductivity but high electrical conductivity. The latter, which is under‐explored, can generate electrical energy from thermal fluctuations without the need of large temperature gradients. Coupling the PAAM‐CNT bilayer with PVDF offers the ability to incorporate the piezoelectric effect in harvesting waste heat, resulting in an 86‐fold enhancement in energy conversion compared to PVDF alone. This preliminary demonstration shows the promise of this system for low‐grade thermal energy harvesting.

Many technologies operate under conditions of energy scarcity and thus demand low‐energy driven actuators. This bilayer actuation system operated under new shape‐changing mechanisms offers highly precise, reversible, and low‐energy driven actuation. Since submolecular actuating units are aligned along the CNTs, this bilayer can be scaled down to micro‐ and nano‐scale. This system lays the foundation for soft material‐based robotics and in vivo micro‐implants that can be controlled by near infrared remotely. By exploiting its ultra‐sensitivity to ambient temperature variations, fluctuating thermal energy can be harvested.

## 2 Results and Discussion

### 2.1 Fabrication

**Figure 1:**
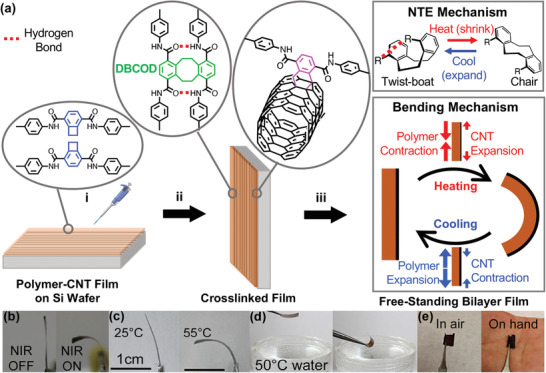
a) Schematic illustration of the bilayer fabrication in three steps: i) beginning with aligned CNTs on a Si substrate, PAAM‐*alt*‐PEG is dropcasted and allowed to dry. The BCB units within the polymer chain are shown (blue); ii) thermal annealing, where both dimerization of BCBs forms DBCOD (green) and covalent linkages form between polymer and CNTs (magenta); and iii) after removing from the substrate, a free‐standing stimuli responsive film is obtained. The polymer exhibits negative thermal expansion (NTE), inducing thermal stress for reversible actuation. The origin of NTE, DBCOD conformational change, is depicted in the upper box. A mismatch in thermal expansion between layers is the origin of bending actuation depicted in the lower box. b–e) optical images of the bilayer film's response to different thermal stimuli, b) before and after exposure to 785 nm light with a total power of 40 mW, c) at 25 and 55 °C heated in an oven, d) before and after placing the film near hot water (50 °C), and e) in air and the same film placed on the palm of a hand.

The fabrication procedure of the bilayer film is displayed in **Figure** **1**a. First, polyarylamide‐co‐polyethylene glycol is dropcasted onto an aligned CNT sheet atop a Si wafer and allowed to air dry. Second, the film is thermally annealed, during which polyethylene glycol (PEG) is removed, the polymer becomes DBCOD‐crosslinked by benzocyclobutene (BCB) dimerization, and covalent linkages are made between the polymer and CNTs. Third, a free‐standing stimuli responsive bilayer (PAAM‐CNT) is obtained by etching the Si substrate.

Polymer synthesis, DBCOD formation, and formation of covalent linkages between polymer chains and the underlying CNT layer can be found in Schemes S1 and S2, Supporting Information. Thermogravimetric analysis (TGA) used to estimate weight percentage of PAAM and PEG as well as atomic force microscopic (AFM) analysis showing polymer self‐assembled morphology facilitating BCB dimerization can be found in the Figures S1 and S2, Supporting Information.

### 2.2 Actuation

Figure 1b–e is a series of optical images to show the response of a set of bilayers to different thermal stimuli, for example, NIR absorption by CNT induced photothermal effect from 34 to 52 °C (Figure 1b), uniform heating in an oven from 25 to 55 °C (Figure 1c), heated from 50 °C water vapor (Figure 1d), and even curling when placed in the palm of a hand (37 °C) (Figure 1e). Movies S1 and S2, Supporting Information, further showcase rapid and large magnitude responses under low‐energy stimuli. Independent of thermal sources, films bent away from the CNT side, indicating that the polymer thermally contracted. This bending direction in response to temperature is opposite to that observed in conventional systems due to fundamentally different bending mechanisms, thermal contraction versus thermal expansion. A new actuation platform that can be driven by a low‐energy input is set forth.

#### 2.2.1 NIR Response

**Figure 2:**
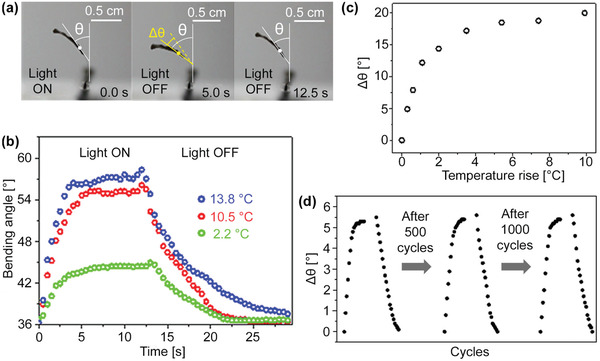
a) A series of optical images of a bilayer film captured before, during, and after laser illumination which resulted in a 10 °C temperature increase. Bending angle, *θ*, defined as the angle between the vertical and the tangent to the bilayer midpoint. b) Bending angle versus time at different laser powers giving 2.2, 10.5, and 13.8 °C temperature rises. c) Change in bending angle versus temperature rise. d) Cycle stability test of photothermal actuation, showing bending behavior under 785 nm laser illumination with a temperature rise of 1.1 °C after 1, 500, and 1000 on/off cycles.

Mechanoresponse was further examined by exposing the new bilayer film to a defocused 785 nm laser. Film temperature was monitored by an infrared camera while film bending was recorded by a digital camera (**Figure** **2**a; Figure S3, Supporting Information). Films bent in the direction toward the polymer side regardless of NIR irradiation on the polymer or CNT side, thus supporting the notion that the polymer undergoes thermal contraction. Despite a low‐energy stimulus, these new bilayer films are able to generate a considerable amount of bending (Figure 1c). To the best of our knowledge, such a large magnitude photothermal response of a solid film, due to thermal contraction and induced by a low‐energy stimulus, has seldom been reported.

The bending angle, *θ*, is plotted as a function of “on” and “off” time (Figure 2b) for three laser power settings to give an average temperature rise of 2.2, 10.5, and 13.8 °C (see Supporting Information for details). Figure 2c shows the change in bending angle, Δ*θ*, as a function of temperature rise from room temp. For a 10 °C temperature rise, Δ*θ* was ≈22°. The response time estimated by Movie S3, Supporting Information, was on the order of milliseconds, comparable if not better than CNTs in paraffin wax on a polyimide substrate.^[^ ^55^ ^]^Furthermore, the bilayer responded to as low as 1.1 °C temperature rises with Δ*θ*  >  5°, showcasing its sensitivity. The observed excellent cycle stability, no visible change after 1000 cycles (Figure 2d), can be attributed to the highly reversible nature of the DBCOD conformational change together with *π*–*π* interactions and covalent linkages between the polymer layer and CNT sheet.

#### 2.2.2 Uniform Heating

**Figure 3:**
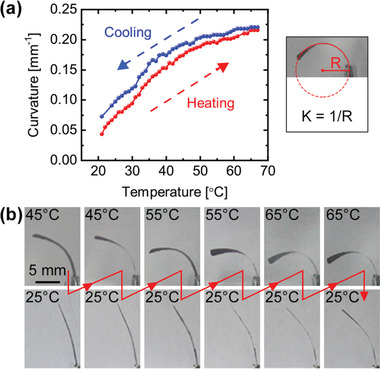
a) Curvature, *K*, of a 1.4 cm‐long bilayer as a function of temperature when heated in an oven. Curvature measured as the inverse of the radius of the circle that matches the arc of the film. b) Images of the film in an oven when repeatedly heated and cooled to set temperatures, with arrows indicating the sequence. Heating to setpoints more than once revealed that the film's response is precise and reversible. 4.7 mm cm^−1^ deflection was observed when heated from 25 to 55 °C.

To quantify thermal response, a bilayer film was subject to a uniform thermal stimulation by heating the entire film in an oven. To cool the film, the oven heater was turned off, and the oven door was gradually opened at various temperatures. Figure S4, Supporting Information, shows heating and cooling rates. **Figure** **3**a shows the film curvature, *K*, analyzed by ImageJ, as a function of temperature. As can be seen, the increase in film curvature gradually levelled off as temperature increased from room temperature up to 65 °C. This set of heating and cooling data indicates the reversible nature of the mechanoresponse of this new bilayer film.

Figure 3b is a series of images of the bilayer film taken after repeated heating and cooling, demonstrating that the degree of bending as a function of temperature is precise. A 1.4 cm‐long film could generate 6.4 mm deflection at 55 °C (a deflection per length of 4.7 mm cm^−1^), on par with the best reported systems.^[^ ^6^, ^16^, ^55^ ^]^ Figure S5, Supporting Information, is a plot of curvature versus time of the film at each set point. The slight change in shape at 25 °C suggests that a bilayer film that experienced higher temperature stimulation requires a longer recovery time to compensate for the moisture depleted at high temperatures as images were taken at equal time intervals.

#### 2.2.3 Directional Bending

**Figure 4:**
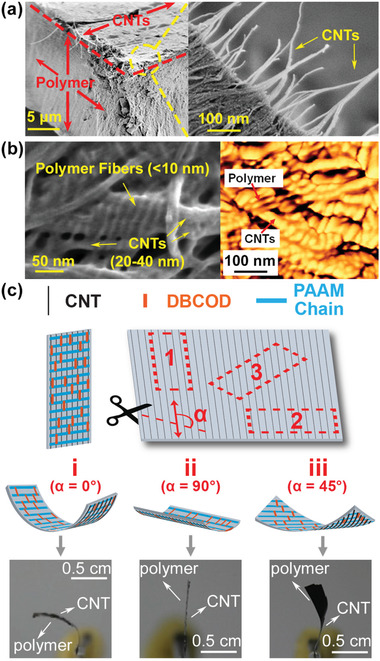
a) Cross‐section and top‐down SEM images of the bilayer film. b) SEM image and AFM height image of the bilayer film, showing alignment of polymer fibers and CNTs. c) Illustration of the alignment of DBCOD units with respect to CNTs and corresponding bending direction upon heating. Polymer chains align perpendicular and DBCODs align parallel to CNTs. Polymer contraction thus occurs parallel to CNTs, and bending, away from CNTs as depicted for three cut angles, i) *α* = 0°, ii) *α* = 90°, and iii) *α* = 45°, relative to CNT alignment. Corresponding optical images of a bending film prepared with cuts (i), (ii), and (iii) are shown.

Scanning electron microscopy (SEM) images (**Figure** **4**a) revealed the bilayer consisted of a thin aligned CNT sheet (0.1 µm) and relatively thick polymer film layer (14 µm). Because of high contrast in properties between rigid PAAM and flexible PEG, polymer chains self‐assembled into nanofibers. Due to *π*–*π* stacking between aromatic rings on PAAM and the CNT surface, which consists of nothing but fused aromatic rings, polymer fibers aligned perpendicular to CNTs. According to AFM height and SEM image analyses (Figure 4b), polymer fibers had a uniform diameter of less than 10 nm whereas the diameters of CNTs were in the range of 20–40 nm. CNT induced polymer self‐assembly has been reported before in which crystallization of polyethylene and nylon 6,6 around CNTs formed a shish‐kebab structure,^[^ ^61^ ^]^ but to the best of our knowledge, the formation of such uniform nanoscale diameter fibers that are well aligned and evenly distributed with respect to CNTs has never been reported. This finding is in corroboration with optical polarization analysis (Figure S6a, Supporting Information). The difference in the index of refraction between two polarizations based on Fourier‐transform infrared spectroscopic analysis (Figure S6b, Supporting Information), further supports the preferential self‐assembly of polymer nanofibers perpendicular to an aligned CNT sheet. In contrast, a pure polymer film without CNTs does not possess preferential optical and submolecular orientation (Figure S7a,b, Supporting Information).

Due to the underlying force imposed by the aligned CNT sheet, DBCOD units are oriented along the CNT backbone. An anisotropic thermal contraction resulting from boat to chair conformational change illustrated in Figure 1a leads to bending toward the polymer side upon heating. Most of the published methods involve isotropic thermal expansion. To investigate the effect of film anisotropy on performance, films were cut vertically, horizontally, and at *α* = 45°, where *α* is defined as the angle between the length of the cut and CNT direction as illustrated in Figure 4c. Figure 4c also shows a set of optical images taken under the same NIR irradiation condition. In case 1 (*α* = 0°), DBCOD units oriented along the length of the bilayer film. The long ends of the bilayer curled toward the polymer upon heating (Figure 4c‐i) due to a large thermal contraction of the polymer film along the DBCOD/CNT direction. For case 2 (*α* = 90°), DBCOD units oriented along the width of the film. As a result, the long ends did not curl. Rather, force was generated along its width, but did not cause buckling (Figure 4c‐ii). Finally for case 3 (*α* = 45°), twisting was observed (Figure 4c‐iii), demonstrating facile pre‐programmability and further corroborating the contention that thermal contraction takes place along the direction of aligned DBCODs and CNTs. Aligned CNT arrays have been used to induce anisotropic bending of a bilayer film before,^[^ ^54^, ^55^, ^62^, ^63^ ^]^ but the ability to generate multiple bending shapes resulting from a CNT induced assembly of conformational change moieties has not been reported before.

This new bilayer utilizes a highly directional thermal contracting polymer film, different from all known bilayer actuation mechanisms. Furthermore, this design could be used in conjunction with the conventional bilayer, whose polymer system offers high thermal expansion, to maximize mechanical bending.

### 2.3 Origin of Low‐Energy Driven Shape Change

#### 2.3.1 Thermomechanical Analysis

**Figure 5:**
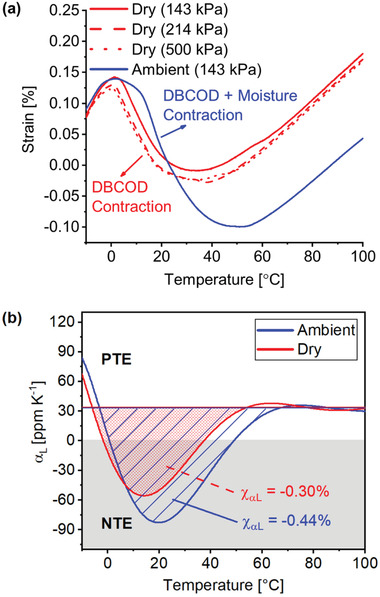
TMA analysis of PAAM‐DBCOD polymer films. a) PAAM‐DBCOD film strain versus temperature after resting in air (ambient) and dry nitrogen (dry) revealing thermal contraction. The constant applied stress is shown in the legend. b) Polymer linear coefficient of thermal expansion as a function of temperature. Films in dry and ambient conditions cross into the negative thermal expansion region from ≈0–35 and ≈0–50 °C, respectively, and were found to exhibit linear thermal expansion capacities of −0.30% and −0.44% when considering the DBCOD contraction component alone.

Thermomechanical analysis (TMA) was conducted to understand the thermal response of PAAM‐DBCOD polymers. **Figure** **5**a shows a comparison of the TMA results of the same sample after storing under ambient conditions (“ambient”) versus after 24 h in dry N_2_ purge (“dry”). The film displayed a normal thermal expansion below 0 °C due to the anharmonicity of atomic oscillations. However, from 0 to 50 °C, abnormal thermal contraction was observed in the “ambient” test. Above 50 °C, this effect disappeared and only thermal expansion was present. Comparing “ambient” versus “dry”, a greater extent of thermal contraction was observed for “ambient.” We posit that moisture in “ambient” solvates the crystalline domain of the polymer, facilitating the DBCOD conformational changes. The noticeably higher thermal contraction for “ambient” is due to the combination of moisture desorption and greater extent of the DBCOD conformational change. It is reasonably assumed that the observed thermal contraction of “dry” is solely caused by the DBCOD conformational change.

In order to rule out a “rubber effect”, that is, an entropy driven thermal contraction observed in stretched rubbers above their glass transition temperature, “dry” was characterized with TMA at different applied loads (Figure 5a). If a rubber effect were present in the films, a greater load would have resulted in greater contraction. However, the independence of the TMA curves on applied stress is evidence that no such effect contributed to this unusual contraction.

To quantify the thermal shrinkage observed, polymer CTE was obtained from TMA data (see Supporting Information for details) and plotted as a function of temperature (Figure 5b). Upon heating, the CTE value became negative and reached a minimum, first for “dry” and then “ambient.” The contraction effect counteracting thermal expansion gradually diminished upon further heating until ≈65 °C, where only thermal expansion remained. These results are correlated with curvature measurements from 20–65 °C (Figure 3a). Further quantifying thermal contraction, we found the net linear NTE capacities, *χ*_*α*L_, the length change observed over the respective 35 and 50 °C NTE temperature ranges for “dry” and “ambient.” The contraction component alone yielded *χ*_*α*L_ = −0.30% and −0.44% for “dry” and “ambient” (see Supporting Information for details).

#### 2.3.2 Differential Scanning Calorimetry

Differential scanning calorimetry (DSC) (Figure S8, Supporting Information) was used to investigate thermodynamic transitions in PAAM‐DBCOD polymer films. Neither glass transition nor melting point was observed from −50 to 200 °C. One large endothermic peak, 208 J g^−1^, was observed for the sample in a pierced pan stored in ambient conditions for more than 10 h. After the measurement, the sample was cooled down to room temperature and rested under dry N_2_ purge for 14 h. There was a small and very broad peak, 3.5 J g^−1^, repeatedly detected.

The amount of enthalpy associated with the DBCOD conformational change (details found in the Supporting Information) within the film is ≈1.1–4.3 J g^−1^ assuming that the DBCOD yield from dimerization is 10–25%. The experimental data falls within the range of the theoretical calculation, using 8–12 kJ mol^−1^ for the DBCOD conformational change from boat to chair, indicating that the small peak in the absence of water was generated by the DBCOD conformational transition in a semi‐crystalline polymer. When the sample was left in air for a few hours, the large endothermic peak quickly restored (Figure S8, Supporting Information), indicating that the observed endothermic absorption was mainly caused by moisture desorption upon heating.

#### 2.3.3 Variable Temperature ^1^H NMR

The dynamic nature of amide‐substituted DBCOD was studied by variable temperature ^1^H NMR (VT‐^1^H NMR). Di‐amide substituted DBCODs were synthesized since this form can be obtained with high purity^[^ ^39^ ^]^ and closely models the DBCOD units in PAAM‐DBCOD. The disappearance of a set of peaks associated with protons on the aromatic rings (*δ* = 6.8–7.7) (Figure S9, Supporting Information) in VT‐^1^H NMR suggested that a portion of DBCOD units underwent a conformational change from one conformer to another ≈50 °C. The contention is supported by the similar changes in amide (*δ* = 9.7–10.1) and methyl (*δ* = 2.0–2.3) regions. The porous nature of annealed aromatic polyamide film may afford enough free volume and mobility to allow the DBCOD conformational change to occur. Despite medium dependence, this set of VT‐^1^H NMR analyses indicates that the DBCOD conformational changes occur at a temperature around and above room temperature.

### 2.4 A Platform for Low‐Grade Thermal Energy Harvesters

**Figure 6:**
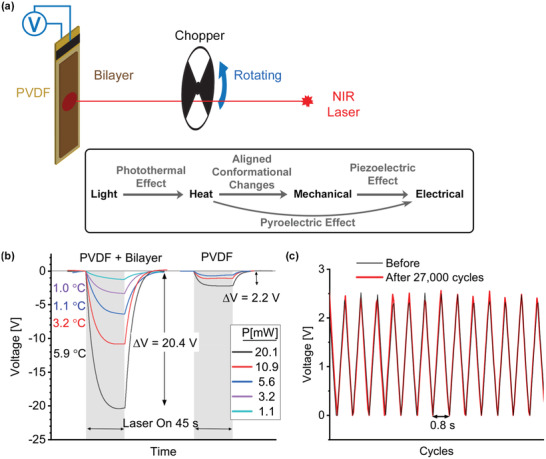
a) Schematic of a NIR light powered AC generator. Open circuit voltage was measured across a bilayer coupled to a piezo‐ and pyroelectric PVDF film while a 785 nm laser was defocused to cover the width of the bilayer film (21 mm x 7 mm) and cycled with a chopper. The energy conversion pathway is displayed in the box. b) Electrical energy harvesting comparison between PVDF alone and the bilayer/PVDF system when exposed for 45 s to different laser powers and corresponding temperatures measured using an infrared camera on the bilayer film. c) AC generation in response to an estimated average temperature rise of 3.3 °C and stability test before and after 27 000 cycles at 1.25 Hz.

We designed a thermally driven AC generator by coupling the bilayer film with a PVDF film. The setup is depicted in **Figure** **6**a in which a NIR source was used to generate heat photothermally. A photo of the device and irradiated area can be found in Figure S10 and Movies S4 and S5, Supporting Information, displaying the device in operation and actuation of the film. When coupled with PVDF, the mechanical bending force generated by thermally triggered DBCOD conformational changes can be converted into electrical energy via the piezoelectric effect. Figure 6b shows the power generation capacity of the PVDF alone in comparison to the bilayer film that is attached to PVDF using a thin layer of wax. For PVDF alone, exposure to NIR led to heating and consequently a voltage gain which was primarily a pyroelectric effect. For the bilayer and PVDF construct, over a nine fold increase in voltage, equivalent to 86 times more electrical energy, was observed under the same NIR irradiation. 20 V could be generated from a temperature rise of the bilayer of ≈6 °C from room temperature. Based on results with and without the bilayer, we estimate that the contribution from piezoelectricity constitutes the majority of the signal for the bilayer‐PVDF construct.

Furthermore, a chopper was used to create a fluctuating thermal source, analogous to thermal waste from exhaust pipelines for example. An infrared camera was used to estimate the average temperature fluctuation (Figure S11, Supporting Information) of ≈3.3 °C. The generated potential by PVDF, open circuit voltage, is plotted in Figure 6c. Not only does this setup effectively convert heat into electrical energy but more significantly, it offers excellent stability at elevated temperature with no obvious degradation after 70 000 cycles using a design to minimize PVDF heating (Figure S12, Supporting Information) or at least 27 000 cycles using a design that can harness both piezoelectric and pyroelectric properties (Figure 6c). The superior cycle stability is due to a combination of the excellent reversibility of the bilayer along with the inherent reversibility of piezoelectricity.

The pyroelectricity of PVDF has been exploited for low‐grade thermal energy harvesting.^[^ ^59^, ^64^ ^]^ However, pyroelectric based energy harvesting offers limited cycle stability. The ability to employ piezoelectricity to harness energy from small thermal fluctuations into electricity has never been reported before to the best of our knowledge.

Figure S13a, Supporting Information, shows the voltage induced by mechanical force generated in the bilayer film with and without moisture. To remove the moisture, a dried molecular sieve was placed in a sealed container containing a bilayer‐PVDF device for an extended period of time. The fact that the data taken before and after 19 h drying via molecular sieve is almost identical suggests that moisture absorption and desorption via temperature fluctuation at 1.25 Hz does not contribute to the observed AC signals. The observed AC voltage gains were mainly generated by the DBCOD conformational changes. Figure S13b, Supporting Information, shows that the thin adhesive layer between the PVDF and DBCOD films has no effect on performance.

Designing an electronic circuit for low‐power generation is a research topic itself.^[^ ^65^, ^66^, ^67^, ^68^ ^]^ For subsequent publications, we will evaluate different circuit designs and further examine energy harvesting efficiency using the bilayer with optimized material properties. PVDF‐based thermal energy harvesting has previously been explored, but the ability to convert low‐grade thermal energy into electricity by employing the piezoelectric (primary) and pyroelectric (secondary) effect sets this system apart from existing systems.

## 3 Conclusion

We have created a new type of low‐energy triggered actuator based on the submolecular actuating units DBCODs, that are self‐assembled in parallel to aligned CNTs. Macroscopic anisotropic thermal contraction, originating from concerted DBCOD submolecular conformational changes, is low‐energy driven and reversible. The low‐energy driven nature is demonstrated by the considerable deformation achieved when placing the bilayer onto one's palm. Gradual curling on one's palm, exposed to heat and moisture from the body, indicates the occurrence of additional conformational changes, facilitated by moisture absorption. This is corroborated by the increased thermal contraction observed in a sample stored in an ambient condition for over 24 h, in comparison to a completely dried sample. Nevertheless, both TMA and PVDF stress analyses support that DBCOD conformational change can take place without moisture.

The polymer shape change induces the bending of the bilayer structure due to a thermal mismatch between the polymer film and underlying aligned CNTs. The anisotropy of the submolecular changes in this system potentially enables shape‐controlled bending at high precision. Further, the bending direction of the new bilayer is opposite, thus complementary, to conventional systems which rely on a material that offers a large positive thermal expansion, therefore adding a new dimension to the existing toolbox.

Compared to PVDF alone, we observed a nearly 86‐fold increase in thermal energy harvesting by coupling the bilayer with PVDF, using an identical energy source. This significant enhancement is due to piezoelectricity resulting from the bending force produced by the DBCOD conformational change upon thermal stimulation. Extended cycle stability has not been reported for pyroelectric‐based thermal energy harvesting devices. In contrast, excellent cycle stability of piezoelectricity together with the inherent reversibility of the bilayer actuation, bestows this new type of AC generator with excellent cycle performance, over 70 000 cycles. Coupled with the bilayer's thermal to mechanical energy conversion in response to few‐degree thermal fluctuations above room temperature, this platform opens a pathway to harvest low‐grade thermal energy.

A low‐energy driven actuator, hitherto unavailable, enables facile and controlled mechanical deformation in shape and magnitude with excellent reversibility at the macro‐, micro‐, and nano‐ scale. This new actuation concept could address key technological challenges in biomedical devices, soft robotics, and other applications where preserving energy is critical, such as in outer space, or those that absolutely require low‐energy driven actuators.

## 4 Experimental Section

### Synthesis and Characterization of Multiblock Polyarylamide

4,4′‐oxydianiline (4,4′‐ODA, 99%) and anhydrous *N*‐methyl‐2‐pyrrolidone (NMP) were purchased from Sigma‐Aldrich and used as received. XTA, a terephthaloyl chloride derivative with a benzocyclobutene group, was provided by Gary Spilman. Amine terminated polyethylene glycol (H_2_N‐PEG‐NH_2_, *M_n_* = 2000) was purchased from Aldrich and used without any purification. Other reagents and solvents were used as received from Aldrich. The synthesis of multiblock polyarylamide was conducted according to the authors′ earlier publication.^[^ ^36^, ^37^ ^]^ TGA (TA instruments SDT Q series) was performed (under N_2_ at a heating rate of 20 °C min^−1^) to obtain the weight percentage of PAAM and PEG.

### Pre‐Treatment of Silicon Substrates

Silicon substrates coated with 100 nm of thermally grown oxides were cleaned by treatment with a freshly prepared “piranha” solution (3/1, *v*/*v*, concentrated H_2_SO_4_/30% aqueous H_2_O_2_) at 100 °C for 1 h and then rinsed with deionized water. All silicon substrates were first treated before use.

### Preparation of Aligned CNT Arrays on Silicon Substrates

Spinnable multi‐walled CNT arrays were synthesized by chemical vapor deposition and then were drawn out on the pre‐cleaned silicon substrates to prepare the aligned CNT arrays with different layers. The typical dimension of CNT was ≈20 nm. Detailed fabrication methods were reported in literature reports.^[^ ^55^ ^]^

### Fabrication of Bilayer Film

After dissolving polymers in NMP, a 300 µL polymer solution (40 mg ml^−1^) was drop‐cast on the multi‐walled CNT substrates (4 cm^2^). After 4–5 days of drying at room temperature, the films were annealed under argon to produce a bilayer film. Thermal annealing in a tube oven, which included an 8 h thermal treatment under argon at 320 °C followed by a 12 h treatment at 350 °C, was applied to prepare a relatively flat bilayer film with covalently linked layers and generate DBCOD. The bilayer films were peeled off from the silicon substrate using buffered hydrofluoric acid to etch the SiO_2_.

### Preparation of Pure Polymer Film

The film was prepared in the same way as a bilayer, except that the solution was drop cast onto silicon substrates that did not contain a CNT array.

### Characterization of Bilayer Film

AFM (XE‐100, Park system) was used to observe the morphology of films before and after annealing. SEM images were performed on Zeiss Gemini 500 electron microscope. Polarizing optical microscope (POM) images were obtained with a Leitz POM.

### Fourier‐Transform Infrared Spectroscopy

All FTIR spectra were acquired on a Jasco 4700 FTIR under ambient humidity. The samples were held between two metal plates with a 3 mm hole in the center. The polymer side was facing the IR beam. The incident IR polarization was controlled by a wire grid polarizer. The FTIR data was collected with 8 cm^−1^ resolution and 325 scan average. The CO_2_ and H_2_O absorption lines were minimized using the Jasco reduction algorithms. Background removal and difference spectra calculation were performed using an asymmetric least squares algorithm.

### Near IR Response Measurement of the Bilayer Film

The setup of thermo‐response measurement via near infrared light is shown in Figure S3, Supporting Information. The bilayer films were fixed at one end for an effective length of 1.5 cm. The distance between the laser and film was set as 5 cm. The heated area was ≈0.4 cm × 0.4 cm. An infrared camera was used to obtain thermal images.

### Thermal Response Measurement of the Bilayer Film

Thermomechanical analysis was carried out on an TA thermomechanical analysis instrument equipped with a tension measurement system. The applied bidirectional force was adjusted based on sample dimensions so that the cross sectional stress was equivalent for different films. All measurements were done under a dry nitrogen atmosphere with a heating rate of 2 °C min^−1^. The first measurement of a film was performed after being stored in ambient conditions for an extended period, called “humid.” After the sample was naturally cooled back down to room temperature and followed by an additional 24 h purge under dry nitrogen, a second measurement was performed, called “dry.” This sequence was carried out at different applied stresses (143, 214, and 500 kPa).

### DSC

A DSC (Netzsch Polyma 214) was calibrated for temperature and sensitivity using adamantane, bismuth, tin, zinc, and indium standards covering the full range of the instrument. All corrected onset temperatures differed from nominal values by less than 0.1 °C, and all experimental sensitivity values differed from the theoretical calculated values by less than 2% for the 5 calibration standards. PAAM‐DBCOD polymers were tested in pierced aluminum pans under nitrogen with a heating rate of 10 K min^−1^.

### NMR

DBCOD monomer (2,3,8,9‐tetramethyl‐1,10‐diphenylamide substituted DBCOD; Figure S9, Supporting Information) was synthesized using the published^[^ ^39^ ^]^ method and dissolved in C_2_D_2_Cl_4_/DMSO‐d_6_ = 1/2 (*v*/*v*) at a concentration of 3 mg mL^−1^ in preparation for VT‐^1^H NMR. The VT‐^1^H NMR spectra were recorded on a Bruker Avance500 II spectrometer. Tetramethylsilane (TMS) was used as an internal standard with an applied temperature coefficient of −5 × 10^−4^ ppm K^−1^ as per the IUPAC's recommendation.^[^ ^69^ ^]^ Peak identification was guided by integration and DFT predictions.

### Piezoelectric Measurement

The AC generator consisted of a 28 µm thick, poled, uniaxial, gold‐covered PVDF film (Precision Acoustics) ≈30 mm x 10 mm, a piece of DBCOD‐CNT bilayer ≈21 mm x 7 mm, and wax adhesive. The sample was covered with a glass beaker to isolate it from air flow. A 785 nm laser was defocused to fill the width of the film (exposure area ≈0.44 cm^2^ as estimated by ImageJ, Figure S10, Supporting Information). For the experiments shown in Figure 6b, the laser was attenuated with different neutral density filters and the power was measured at the sample with a power meter (Thorlabs PM100D with a Thorlabs S121C photodiode power sensor). An infrared camera (Fluke TiS10) was used to obtain thermal images. To generate AC signals, a chopper was placed in between the film and laser source. Open circuit voltage measurements were taken with a BioLogic potentiostat. Data were baseline subtracted, translated, and overlaid.

### Statistical Analysis

Representative data were presented as raw data except for open circuit voltages which were baseline subtracted and shifted to 0 V for comparison.

## Conflict of Interest

The authors declare no conflict of interest.

## Supporting information

Supporting Information

Supplemental Movie 1

Supplemental Movie 2

Supplemental Movie 3

Supplemental Movie 4

Supplemental Movie 5

## Data Availability

Data available on request from the authors.
