# Supplementary material for: Directional, Low‐Energy Driven Thermal Actuating Bilayer Enabled by Coordinated Submolecular Switching
Source: Adv Sci (Weinh). 2021 Oct 23;8(23):2102077. doi: 10.1002/advs.202102077 (PMC8655216; doi:10.1002/advs.202102077)
Supplement: Supplementary file 1 — Supporting Information [file ADVS-8-2102077-s005.pdf]

ADVANCED SCIENCE
Open Access

Supporting Information

for *Adv. Sci.,* DOI: 10.1002/advs.202102077

Directional, Low-Energy Driven Thermal Actuating Bilayer Enabled by Coordinated Submolecular Switching

*Michael Leveille, Xinyuan Shen, Wenxin Fu, Ke Jin, Muharrem Acerce, Changchun Wang, Jacqueline Bustamante, Anneka Miller Casas, Yuan Feng, Nien-Hui Ge, Linda S. Hirst, Sayantani Ghosh, Jennifer Qing Lu**

Supporting Information

Directional, Low-Energy Driven Thermal Actuating Bilayer Enabled by Coordinated Submolecular Switching

*Michael Leveille, Xinyuan Shen, Wenxin Fu, Ke Jin, Muharrem Acerce, Changchun Wang, Jacqueline Bustamante, Anneka Miller Casas, Yuan Feng, Nien-Hui Ge, Linda S. Hirst, Sayantani Ghosh, Jennifer Qing Lu**

Thermal Analysis: In order to determine the coefficient of thermal expansion (CTE), length vs temperature was plotted and best fit with a 9th order polynomial curve fit (R-Square > 0.997) using Origin. The fit was differentiated and divided by film length at each temperature to plot the linear CTE, given by:

$$\alpha_{\mathrm{L}} = \frac{1}{L}\frac{\partial L}{\partial T} \tag{1}$$

where L is the instantaneous length of the film and T is temperature. Furthermore, net negative thermal expansion (NTE) capacity, χ_{α}, originally introduced in terms of isotropic NTE materials as a means of comparing their thermal contraction,[1] can be given for linear NTE materials as:

$$\chi_{\alpha\mathrm{L}} = \int_{T_1}^{T_2} \alpha_{\mathrm{L}} dT = \frac{\Delta L}{L} \tag{2}$$

where T_1 and T_2 bound the domain in which NTE is observed. $\chi_{\alpha\mathrm{L}}$ is simply the net strain over the NTE temperature range. Integrating $\alpha_{\mathrm{L}}(T)$ between its roots, T_1 and T_2, yielded $\chi_{\alpha\mathrm{L}}$ = -0.14% and -0.25% for "dry" and "ambient". The contraction component alone was estimated by setting a baseline at 33 ppm K^{-1} and integrating the area below.

Estimating Film Temperature Rise: A typical laser beam is usually regarded as a Gaussian beam, so the peak intensity is twice the average intensity.[2-3] The peak intensity, I_p, is found at the center of the beam and given by

$$I_p = \frac{2P_0}{\pi w^2} \tag{3}$$

where P_0 is the total power and w is the beam waist, defined as the radial distance from the beam axis where the intensity is I_p/e^2. Therefore more than 86% of the power is within the beam waist. The average intensity, I_{avg}, can be estimated as

$$I_{avg} = \frac{P_0}{\pi w^2} = \frac{1}{2} I_p \tag{4}$$

the total power divided by the area of the beam within the waist, equal to half the peak intensity. Furthermore, power is directly proportional to intensity, and temperature rise is directly proportional to power (Figure S14, Supporting Information). Therefore, the peak temperature rise (measured by an infrared camera) is twice the value of the average temperature rise. For example, when room temperature is measured at 24.3 °C and peak temperature measured as 28.6 °C, the average temperature rise was calculated as (28.6 - 24.3) * 0.5 = 2.2 °C.

Measuring DBCOD Conformational Change with DSC: The enthalpy of DBCOD conformational change in a PAAM-DBCOD polymer, $\Delta H_{PAAM\text{-}DBCOD}$, can be estimated by:

$$\Delta H_{PAAM-DBCOD} = \frac{x * \Delta H_{DBCOD}}{Mw} \tag{5}$$

where x is the molar fraction of repeat units that formed DBCOD, ΔH_{DBCOD} is the enthalpy associated with the DBCOD conformational change, and M_W is the molecular weight of the repeat unit (717 g mol^{-1}). According to DFT, the enthalpy associated with the DBCOD conformational change is 2 - 3 kcal mol^{-1} (8.4 - 12.6 kJ mol^{-1}).[4] The estimated yield for DBCOD during thermal annealing is about 10 - 25%. This DBCOD transition would therefore produce 1.1 - 4.3 J g^{-1}.

Electrical Energy of the AC Generator: The relationship between generated voltage and energy of the reported AC generator can be seen from Equation (6) and (7) for a pure resistive circuit:

$$E = \int p(t)dt = P_{avg}\Delta t \quad (6)$$

$$P_{avg} = I_{rms}V_{rms} = \frac{1}{2}I_0V_0 \quad (7)$$

where E is energy, $p(t)$ is instantaneous power, P_{avg} is average power, Δt is change in time, I_{rms} is the root mean square current, I_0 is current amplitude, V_{rms} is the root mean square voltage, and V_0 is voltage amplitude. Additionally, in a simple circuit with voltage, V, current, I, and resistance, R, the current is directly proportional to the voltage as stated by Ohm's law. Thus, a 9.3 fold increase in voltage with the addition of the PAAM-CNT bilayer to PVDF theoretically leads to a 9.3, 86.5, and 86.5-fold increase in current, P_{avg}, and E.

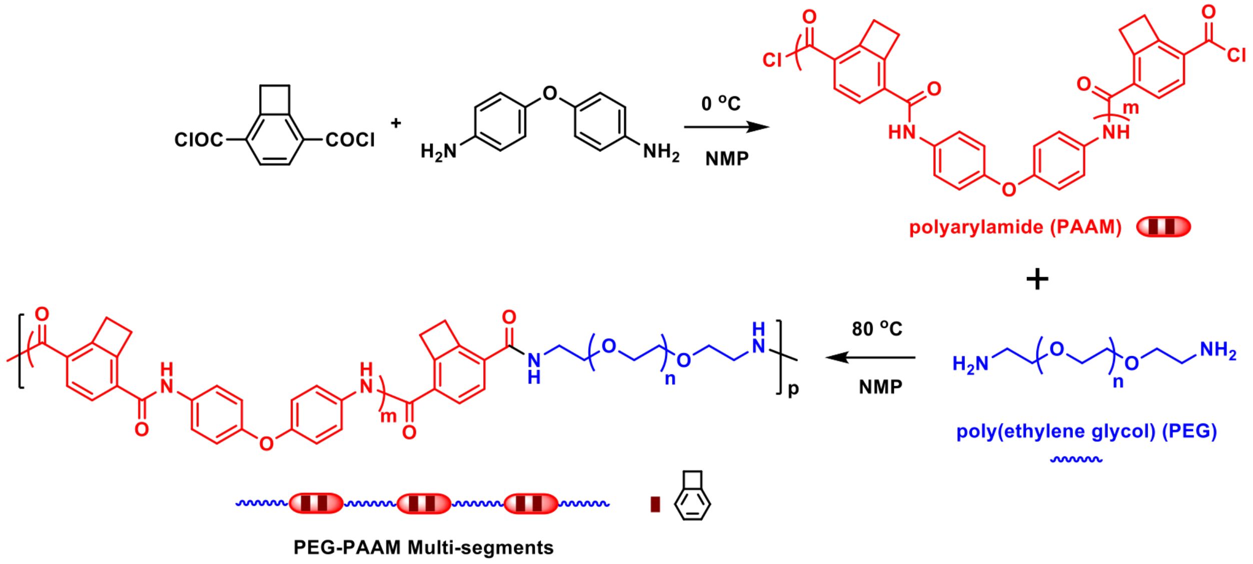

Scheme S1. Synthesis scheme of multi-segment PAAM-*alt*-PEG

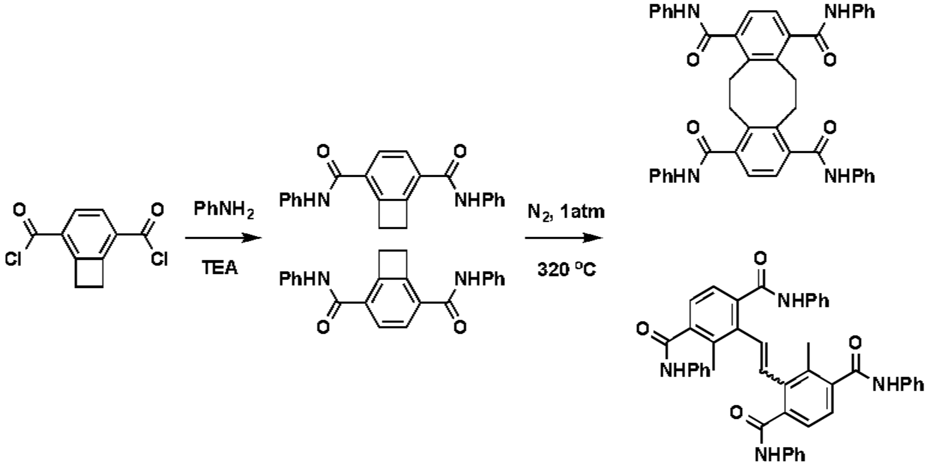

Scheme S2. Benzocyclobutene (BCB) dimerization. One of the products is DBCOD.

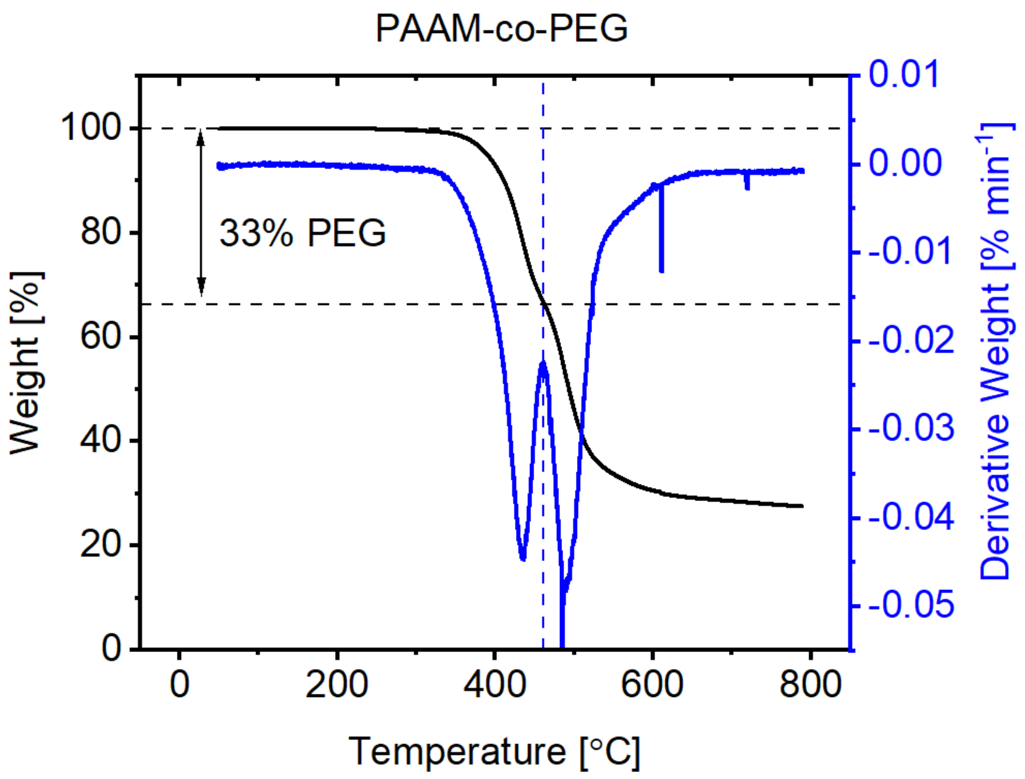

Figure S1. Thermogravimetric analysis of multi-segment PAAM-*alt*-PEG under nitrogen and heating rate of 20 ºC min^{-1}. The derivative weight % is shown to distinguish degradation of PAAM and PEG, indicating about 33 wt% PEG.

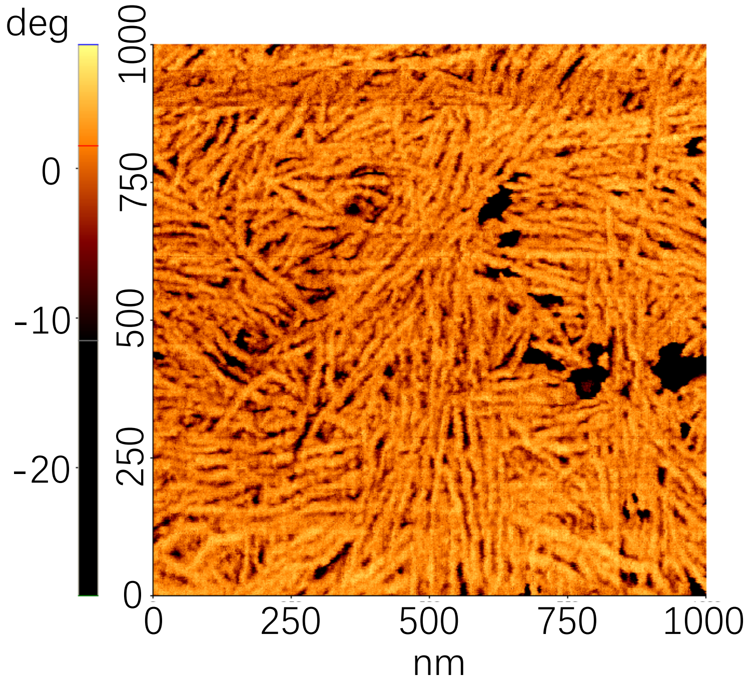

Figure S2. AFM phase image of pre-annealed PAAM-*alt*-PEG

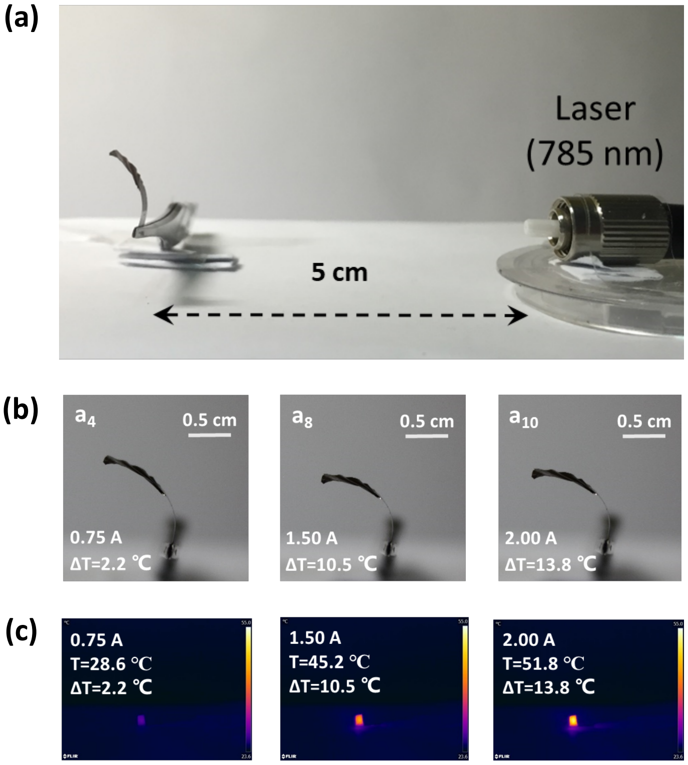

Figure S3. a) NIR setup, laser was defocused so that the entire width of the film was exposed. b) A series of images showing the bending behavior of a bilayer film with different temperature rises by adjusting the current value of a 785 nm laser. c) Corresponding infrared thermal images showing the temperature rise of the bilayer film. Average temperature rise was estimated according to a gaussian beam (see Supporting Information, section "*Estimating Film Temperature Rise"*).

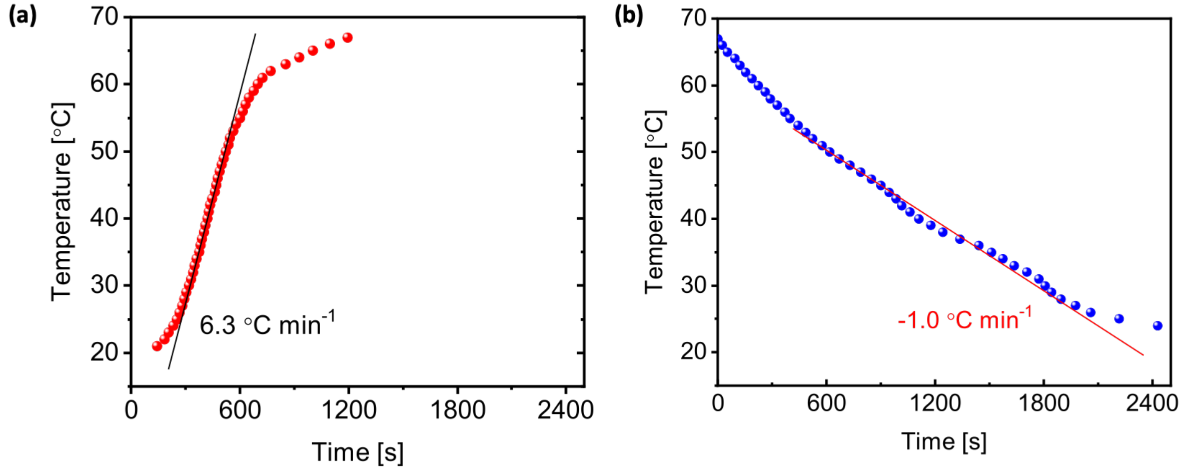

Figure S4. Temperature vs. time during a) heating and b) cooling of a PAAM-CNT bilayer in an oven. Solid lines are guides for the eye with the corresponding heating rates shown. During cooling, the chamber was slightly opened twice before being fully opened around 35 ºC.

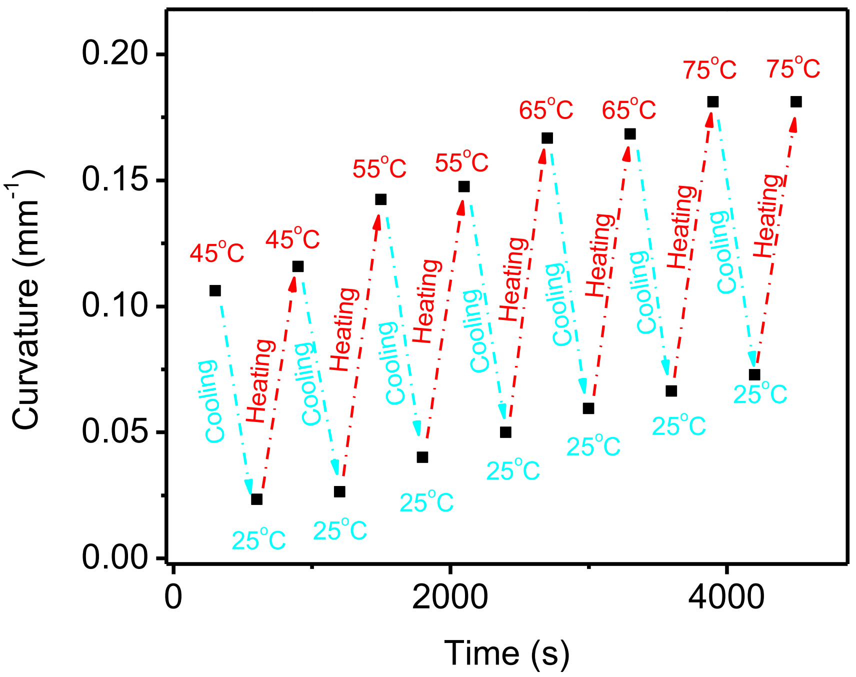

Figure S5. Curvature vs. time at various temperature setpoints of a PAAM-CNT bilayer repeatedly heated and cooled in an oven. Increase in curvature at 25 ºC suggests that the film needs more time to recover from moisture depleted when cooling from higher temperatures. Measurements were taken at equal time intervals.

(a)

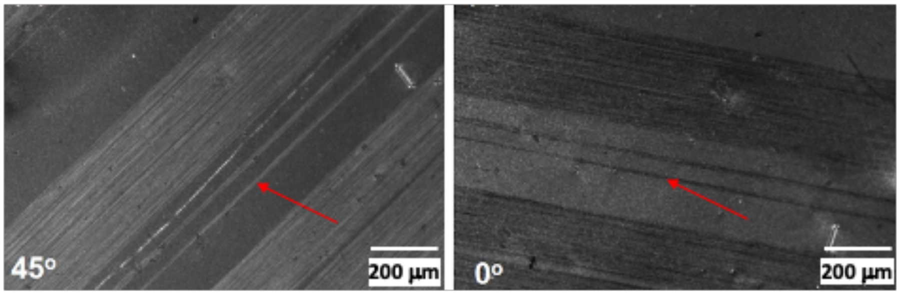

(b)

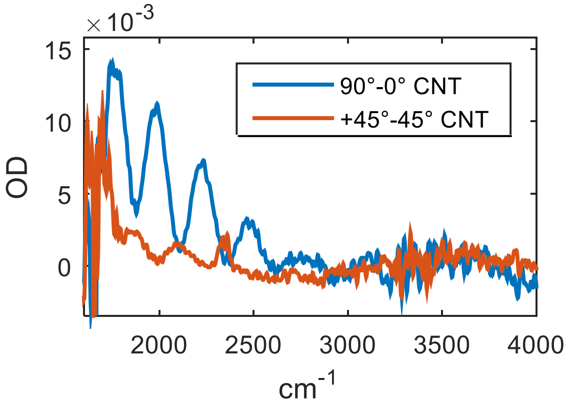

Figure S6. Bilayer Anisotropy. a) Polarized optical images of a bilayer film with arrows to guide the eye. Transmission at 0º and 45º indicate alignment of polymer fibers and CNTs, respectively. b) Difference FTIR spectra revealing birefringence of a bilayer film: (blue) the difference between the two spectra taken with the IR polarization set to 90º and 0º with respect to the CNT axis; (red) the difference between spectra taken with the polarization set to 45º and –45º with respect to the CNT axis. The strong oscillations shown in the blue difference spectrum result from the difference in the index of refraction between the two polarizations. Some oscillations can still be seen in the red difference spectrum due to small misalignment of 0º polarization from the CNT axis, as performed visually. The small peak at around 2300 cm^{-1} comes from CO_2 vapor.

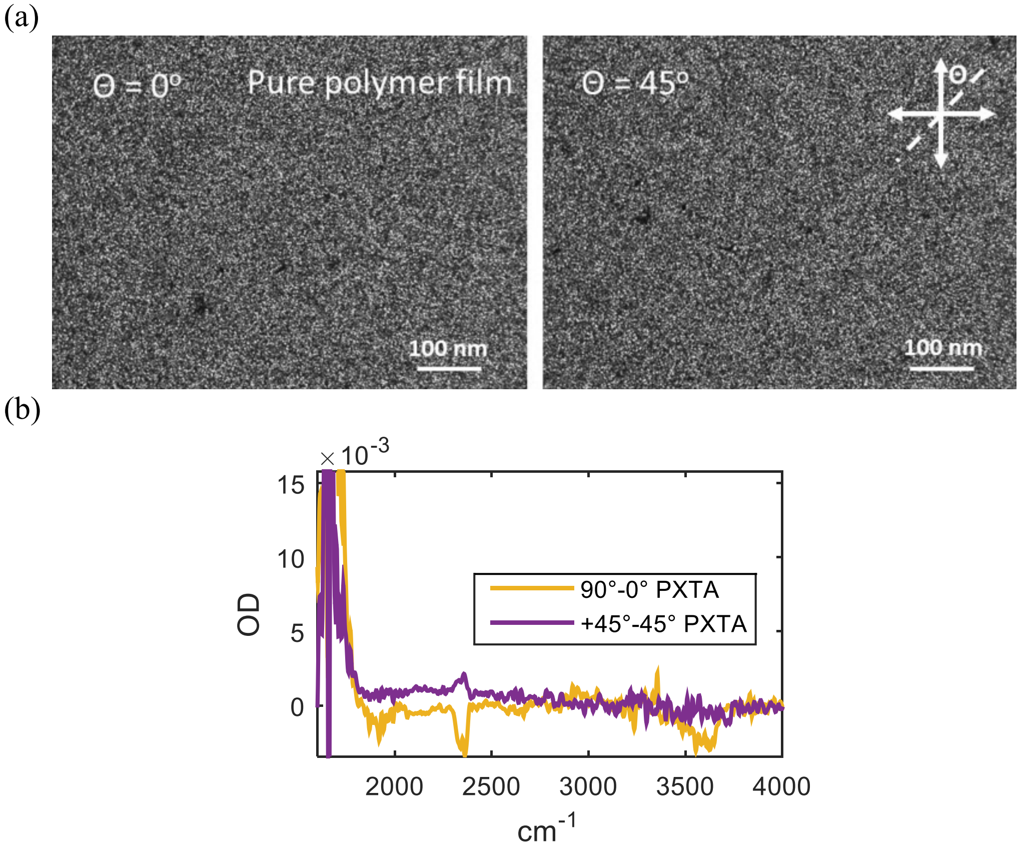

Figure S7. a) Polarized optical images of pure polymer film. b) Difference FTIR spectra revealing isotropy of pure polymer film: (gold) the difference between the two spectra taken with the IR polarization set to 90º and 0º with respect to one chosen axis; (purple) the difference between spectra taken with the polarization set to 45º and -45º with respect to the same axis. The peak at around 2300 cm^{-1} comes from CO_2 vapor.

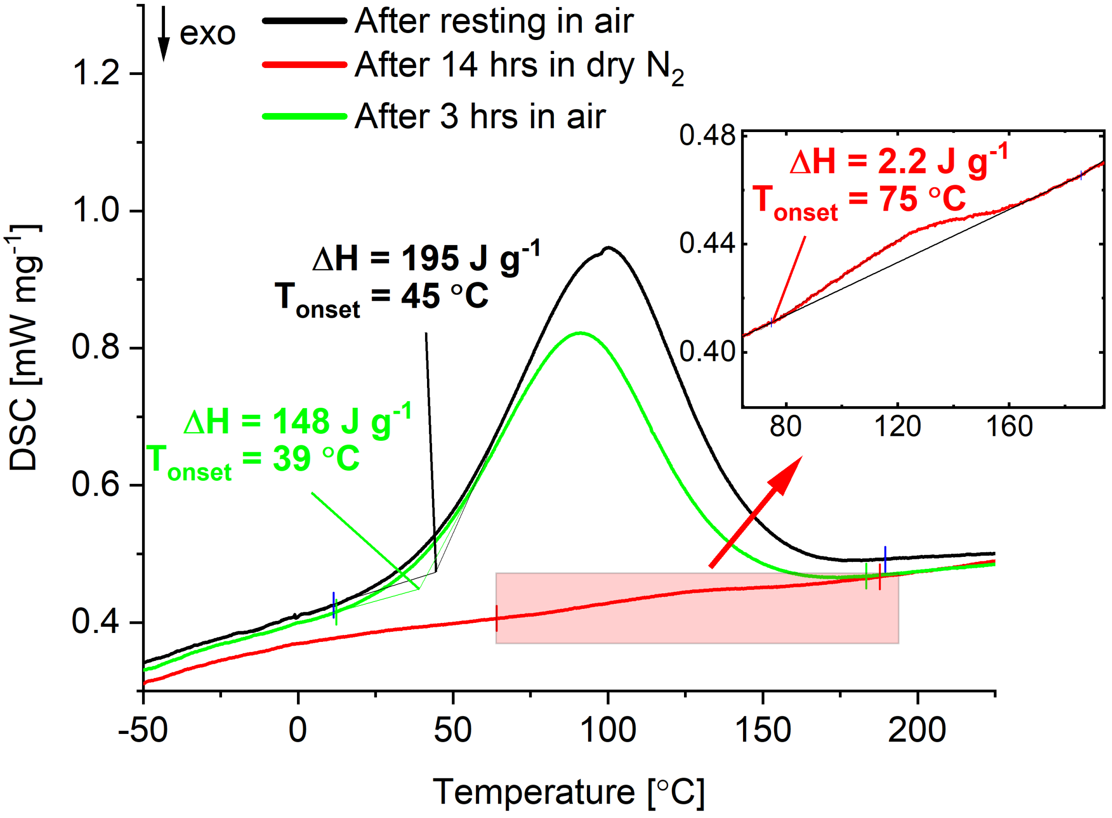

Figure S8. DSC of PAAM-DBCOD under nitrogen atmosphere in a pierced aluminum pan and heating rate of 10 °C min^{-1} after resting in air (black), resting in dry nitrogen for 14 hrs (green), and resting in air for 3 hrs (red).

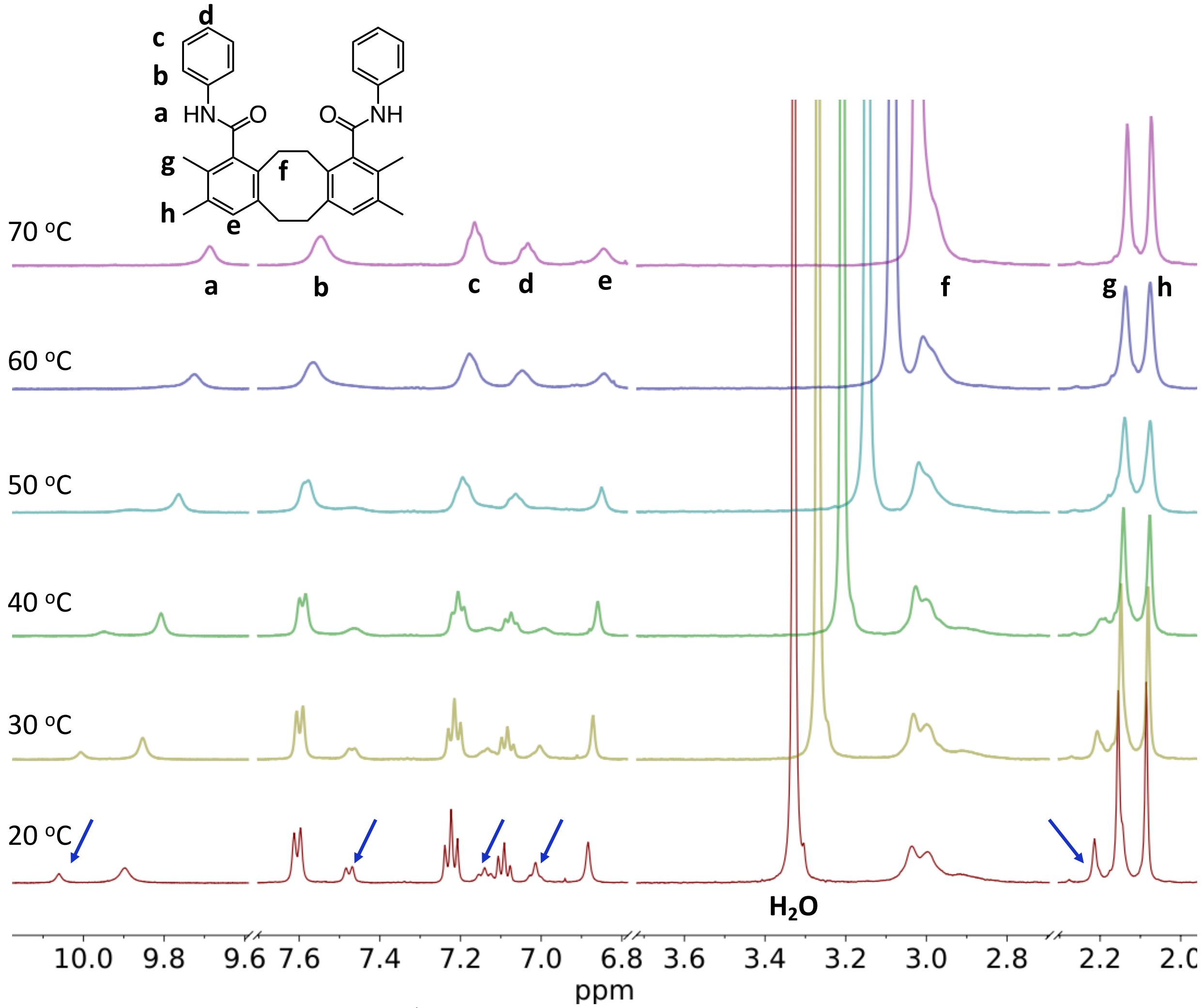

Figure S9. Variable temperature ^{1}H NMR of diphenyl amide substituted DBCOD monomer in $C_2D_2Cl_4$/DMSO-d_6 = 1/2 (v/v). Proton signals a-h were labeled on spectrum. The set of peaks (highlighted with arrows) that disappeared with temperature indicated DBCOD conformational transitions.

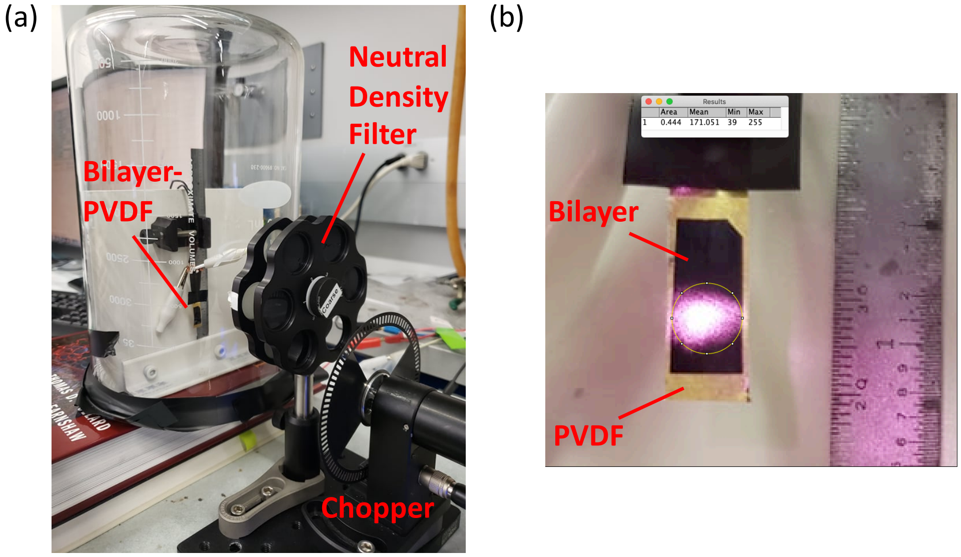

Figure S10. a) Photograph of the energy harvesting setup. The NIR source is located 30 cm from the film. Power was measured at the film. A beaker was placed over the device to insulate it from air flow, b) ImageJ analysis indicating an irradiation area of the bilayer of about 0.44 cm^2.

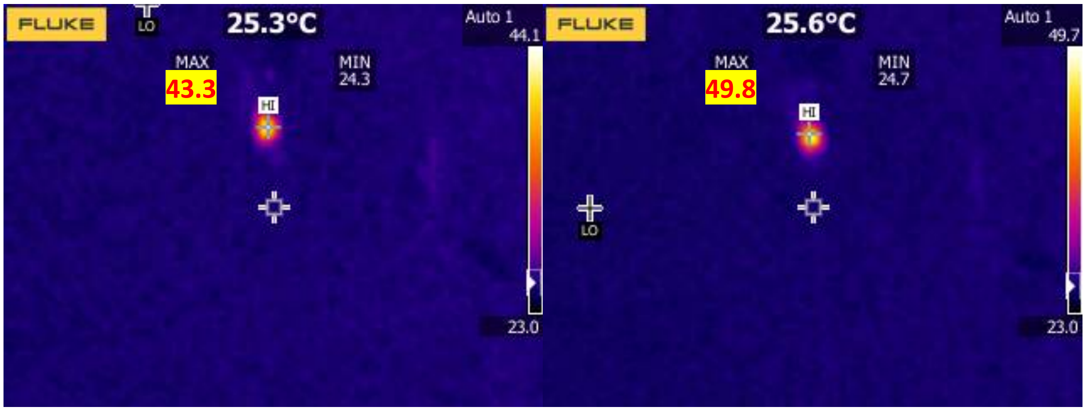

Figure S11. Thermal images of the bilayer-PVDF construct during 1.25 Hz exposure to 77.3 mW of defocused 785nm laser. The average temperature fluctuation is estimated to be about 3.3 °C. Average temperature rise was estimated according to a gaussian beam (see Supporting Information, section "*Estimating Film Temperature Rise*").

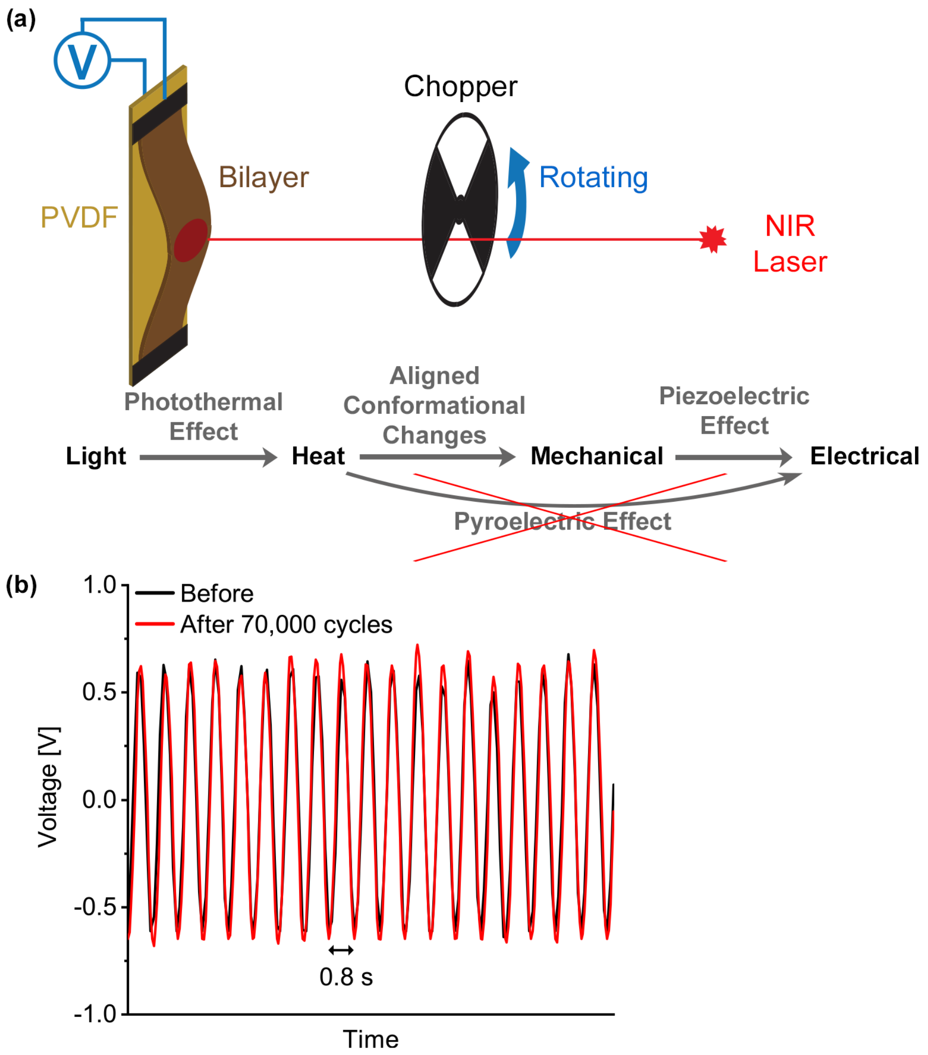

Figure S12. a) Alternative setup of AC generator isolating piezoelectric energy conversion. b) Open circuit voltage output offering excellent stability with no obvious degradation after 70,000 cycles at moderate power (1.25 Hz, 77.3 mW)

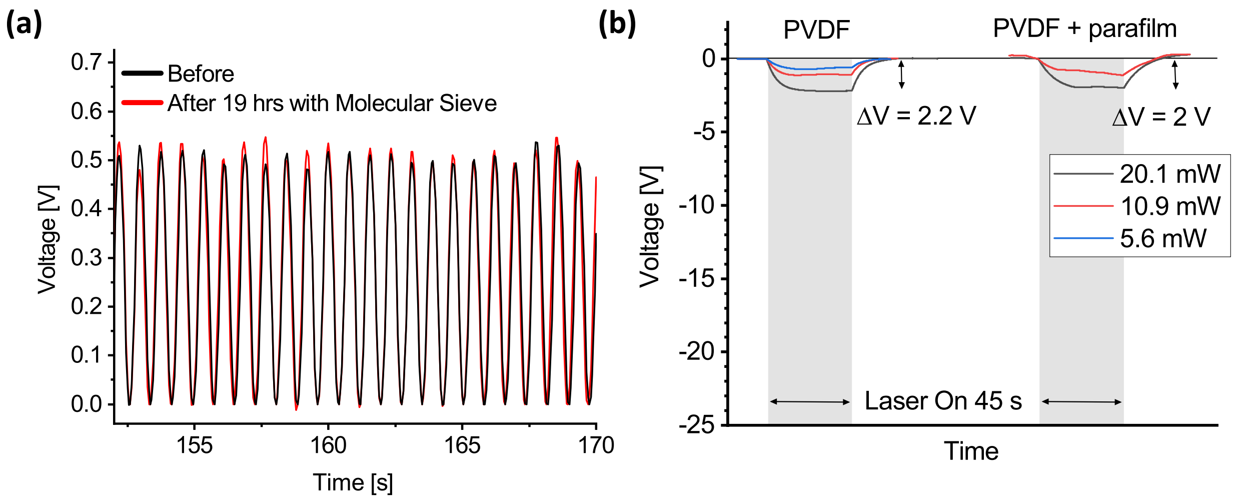

Figure S13. a) Open circuit voltage of an AC generator under ambient and dry conditions. b) Electrical energy harvesting comparison between PVDF alone and PVDF with a thin piece of wax (used as the adhesive layer in the bilayer/PVDF system) when exposed for 45 s to different laser powers.

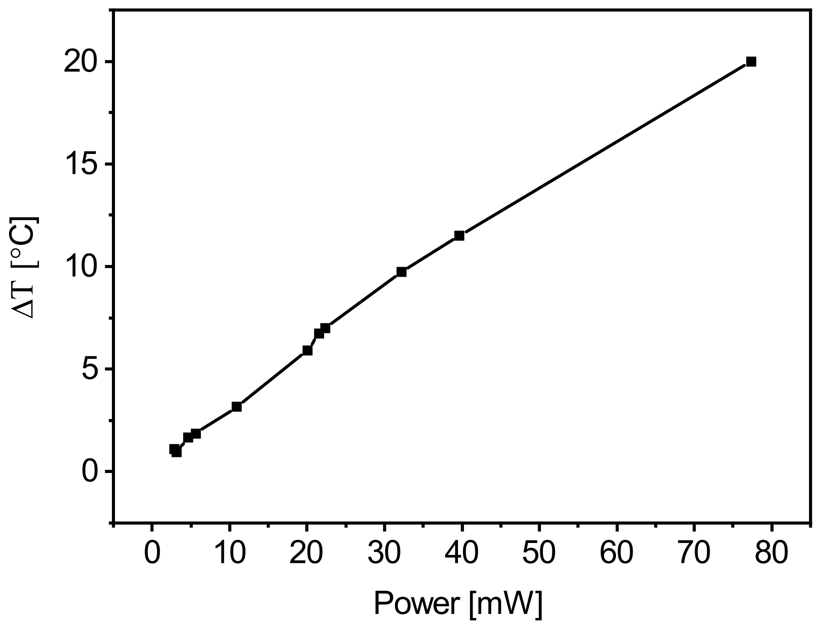

Figure S14. Temperature rise vs power of the bilayer-PVDF construct when exposed to a 785 nm laser source. Temperature rise is directly proportional to power.

References

[1] C. S. Coates, A. L. Goodwin, *Mater. Horiz*. **2019**, *6*, 211.

[2] F. M. Dickey, S. C. Holswade, D. L. Shealy, *Laser Beam Shaping Applications,* CRC Press, Boca Raton, FL. 2006, Ch.5.

[3] W. Demtröder, in *Laser Spectroscopy 1: Basic Principles*, (Ed: W. Demtröder), Springer Berlin Heidelberg, Berlin, Heidelberg 2014, Ch.7.

[4] X. Y. Shen, C. Viney, E. R. Johnson, C. C. Wang, J. Q. Lu, *Nat. Chem*. **2013**, *5*, 1035.
